# Impossibility results about inheritance and order of death

**DOI:** 10.1371/journal.pone.0277430

**Published:** 2022-11-10

**Authors:** Yue Wang

**Affiliations:** 1 Department of Computational Medicine, University of California, Los Angeles, California, United States of America; 2 Institut des Hautes Études Scientifiques, Bures-sur-Yvette, Essonne, France; Teikyo University, School of Medicine, JAPAN

## Abstract

If several relatives died with no will, the order of their deaths could affect the inheritance result. When the order of death is unknown, there are three approaches to determine the inheritance result in this simultaneous death situation: apply an inheritance method that is not affected by the order of death; artificially assign the order of death; stipulate that persons with unknown orders do not inherit each other. The last approach is adopted by the current French Civil Code (denoted as the French Approach). We prove that under some basic requirements, the French Approach is the only valid solution to the order of death problem. Therefore, the inheritance law of a country that does not adopt the French Approach either has unsolvable inheritance problems or violates basic requirements. In the appendix, we study the existence and uniqueness of inheritance methods that are invariant for different orders of death and only violate one requirement, such as gender equality.

## 1 Introduction

If a person died without a will (intestate), her/his property shall be divided according to the inheritance law. At the death of a person A, all relatives who are **alive** at this time point shall be considered in the inheritance, while relatives who died before A shall be excluded. An **inheritance method** describes which living relatives can inherit, and how much each relative should inherit. For example, in China, after the death of a person Alice, her property shall be divided equally by her living parents, spouse, and children.

A problem emerges **when the order of death is unknown** (e.g., A and B died in the same event). In this scenario, we do not know whether A is already dead at the death of B (so that A should be excluded from the inheritance of B), and **the inheritance cannot be carried out**. In this paper, we will discuss different approaches to this problem.

The first approach is to pick an order of death (A died before B or after B) arbitrarily. This approach does not work for many inheritance methods. For example, in China, if Alice died after her parents, Alice’s siblings could not inherit her property; if Alice died before her parents, then her parents could inherit some of Alice’s property, which would then be inherited by Alice’s siblings. Therefore, **the order of death can affect the inheritance result**. To make this approach work, we need to construct a **commutative** inheritance method, meaning that it does not depend on the order of death. However, we shall prove that under some basic requirements, an inheritance method cannot be commutative.

The second approach is to adopt an inheritance method that is not commutative. When the order of death for A and B is unknown, and the order determines the inheritance result, an order of death is **artificially assigned** by given rules. During 1804-2002, by the French Civil Code, one can assign that either A died before B (denoted as A < B) or A died after B (denoted as A > B), based on age and gender. With this assignment, we obtain a strict total order for all dead persons (e.g., A < C < B < D). In the current Chinese Civil Code, besides A < B and A > B, one can also assign that A and B died simultaneously (denoted as A = B), so that each is excluded from the inheritance of the other. With this assignment, we obtain a generalized total order for all dead persons (e.g., A = D < C = B). Nevertheless, if multiple persons died, and the order of death is partially unknown, then such artificial assignments might lead to contradictions. We shall prove that under some basic requirements, such contradictions are unavoidable, so that the inheritance results cannot be determined.

The third approach is to directly stipulate that persons with unknown order of death do not inherit each other, so that we avoid assigning an order. In this approach, the death time of A and the death time of B are incomparable (denoted as A ≈ B). With this approach, we only have a partial order, since A ≈ B and B ≈ C do not necessarily imply that A ≈ C. When the order of death is unknown, the current French Civil Code (since 2002) always assigns A ≈ B. Although France is not the first country to adopt this approach, we suppose that France might be the first country to notice the problems of other approaches and switch to this approach. Thus we will call this approach the **French Approach**. The second and the third approaches might be combined, so that we can assign A < B, A > B, A = B, A ≈ B. We shall prove that under some basic requirements, the only valid solution to the order of death problem is purely assigning A ≈ B, namely the French Approach.

In Section 2, we provide an incomplete summary of related laws and works concerning inheritance and the order of death problem. In Section 3, we introduce the setup and some basic requirements for different approaches to the order of death problem. Then in Section 4, we propose rigorous proof that under such basic requirements, all approaches except the French Approach shall fail. This approach of proposing seemingly reasonable criteria and proving incompatibility is similar to our previous work [1]. We finish with conclusions and discussions in Section 5. In the appendix, we show that under more restrictions, there exist four inheritance methods that are commutative, and only violate one requirement, e.g., gender equality.

## 2 Related laws and works

Legislators in many countries have noticed that inheritance might be problematic when multiple persons died and the order of death is unclear (simultaneous death). These countries adopt different approaches concerning the order of death. In some other countries, the inheritance laws do not have related regulations, but researchers have debated on this issue [2].

The inheritance law of Roman Empire states that when parents and children died simultaneously, underage children are presumed to die before parents, and grown-up children are presumed to die after parents [3].

The 1804 version of French Civil Code (Napoleonic Code) determines which person dies first, depending on age and gender. For example, for persons under 15 years old, younger ones are presumed to die first. For adults of the same age, females are presumed to die first [4].

The current version of French Civil Code (since 2002) stipulates that if one cannot determine the order of death for two persons, they do not inherit each other [5].

The General State Laws of the Prussian States and the General Civil Code of the Austrian Empire stipulate that if the order of death cannot be determined, the deceased do not inherit each other [2].

The current version of Chinese Civil Code (since 2021) determines which person dies first or stipulate that two persons die simultaneously, depending on relative relationships. For example, sisters are presumed to die simultaneously, and daughter is presumed to die after mother [6].

In England and Wales, the commorientes rule in the Law of Property Act 1925 states that when dying simultaneously, younger persons are presumed to die before older persons [7].

The Uniform Simultaneous Death Act in the United States regulates that two persons died within 120 hours are excluded from the inheritance of the other [8].

The 2002 version of the Russian Federation Civil Code states that persons died in the same calendar day (calculated by local time) do not inherit each other. The 2016 version adopts a different rule that persons do not inherit each other only if the order of death cannot be determined [9].

There have been many research articles that discuss the inheritance with simultaneous death in the viewpoint of history [2, 10, 11] or jurisprudence [12–17]. However, none of them apply mathematical methods. We use rigorous mathematics to prove that an inheritance law either adopts the French approach, or has situations where inheritance cannot be carried out, or violates some criteria. The order of statutory inheritance is still an issue under debating, and there is no consensus on unification, even within EU [18, 19]. Different law practices prevent adopting the same solution. About the criteria that an inheritance law should follow, there are also many different opinions [20–24]. We just aim at providing mathematical derivations about the order of death problem, as a reference for social scientists and legislators.

## 3 Setup and requirements for inheritance and order of death

To determine the inheritance result when the order of death is unknown, we have different approaches: (1) propose an inheritance method that does not depend on the order of death; (2) introduce a scheme to artificially assign the order of death; (3) always assign A ≈ B (the French Approach). In this section, we introduce the setup and requirements for different approaches.

To simplify the discussion, we only consider biological parents-children relationships, but not adoption. Besides, incest is excluded: a person does not marry with her/his parent, child, sibling, parent’s sibling, sibling’s child (cousin does not matter). This guarantees two facts that will be used later: a person’s parents, paternal grandparents, and maternal grandparents are six different persons; a person’s parent-in-law is not her/his grandparent.

In this paper, ancestors of a person A mean direct ancestors, such as parents, grandparents, great-grandparents, et al. Descendants mean direct descendants, such as children, grandchildren, great-grandchildren, et al. Descendants of ancestors (excluding A, A’s ancestors, and A’s descendants) are collateral relatives, e.g., siblings, aunts, nieces. Blood relatives consist of ancestors, descendants, and collateral relatives. The relative relation between two persons is a chain of the following relations: parent/child, spouse, sibling. We do not distinguish the birth order of siblings.

When we discuss the death of multiple persons, unless otherwise specified, we always assume they died in a very short period of time, so that no change (except inheritance) happens to the property of each person. In other words, we study inheritance in a static way.

### 3.1 Inheritance methods

Consider a person A, A’s parents B and C, B’s parents D and E, C’s parents F and G. Assume they have no other living relatives. F and G are already dead. We consider the property distribution of each person, such as $P=(\begin{array}{ccccccc} \text{A} & \text{B} & \text{C} & \text{D} & \text{E} & \text{F} & \text{G}\\ 100 & 400 & 400 & 50 & 50 & \times & \times \end{array})$, where each number is the amount of property owned by the corresponding person, and “×” means the person is dead. The death of a person and the following inheritance changes the property distribution. For example, by the Civil Code of China, the heritage is equally split by parents, children and spouse. Then the death of B (denoted as $D_{\text{B}}$) has the effect $D_{\text{B}}P=(\begin{array}{ccccccc} \text{A} & \text{B} & \text{C} & \text{D} & \text{E} & \text{F} & \text{G}\\ 200 & \times & 500 & 150 & 150 & \times & \times \end{array})$, if we do not consider inheritance tax.

We can see that each $D_{\text{A}}$ maps one property distribution to another. Then we can regard each $D_{\text{A}}$ as an operator from the space of property distributions to itself. To avoid ambiguity, we stipulate that applying $D_{\text{A}}$ when A is already dead has no effect, e.g., $D_{\text{F}}P=P$.

At the death of a person, depending on the relative relationships, an **inheritance method** determines which living relatives can inherit, and what amount of heritage can be inherited. We explicitly state this as a criterion.

**(C0)**: The inheritance method determines a non-random inheritance result that only depends on relative relationships (gender can be considered). Other factors, especially age, shall not be considered.

By (C0), if A’,B’,C’,D’,E’,F’,G’ have the same relative relations with A,B,C,D,E,F,G, then the operator $D_{{\text{A}}^{\prime }}$ has the same effect with $D_{\text{A}}$. In other words, given the relative relations, the inheritance method fully determines the operators.

If we do not have any restrictions on inheritance, one simple solution can avoid the order of death problem: no one can inherit, and the heritage always goes to the treasury. To exclude such trivial inheritance methods, based on (C0), we further propose three rules (R1)-(R3) that specify inheritance according to relative relations.

(R1) The inheritance method satisfies gender equality, meaning that the following pairs shall be equally treated in inheritance: father-mother, son-daughter, brother-sister, husband-wife.
(R2) At one person’s death, all her/his sons, or all daughters, or all children can always inherit.
(R3) At one person’s death, if she/he has no living spouse, descendant, or sibling, then her/his father or mother or both parents can inherit. In the case that both parents can inherit, if both parents are alive, they split all the heritage.

If (R1) is satisfied, then (C0) becomes “the inheritance method only considers gender-neutral relative relationships”; (R2) becomes “all children can always inherit”; (R3) becomes “both parents can conditionally inherit, and when they can inherit and they are both alive, they split all the heritage”.

(C0) and (R1)-(R3) are satisfied in the inheritance laws of many countries, such as China, England, France, and Germany [6, 23, 25]. Historically, inheritance is conducted according to relative relations, and close relatives are more favorable than distant relatives. Thus (C0), (R2), (R3) have been widely practiced. Besides, countries which have gender equity in their constitutions must follow (R1). The second half of (R3) (both parents split all the heritage) might look less natural, but it excludes a trivial (but commutative) inheritance method that all living persons in the world split the heritage equally.

In many inheritance laws, different orders of death can produce different inheritance results. In the above example, we have $D_{\text{C}}\left(D_{\text{B}}P\right)=(\begin{array}{ccccccc} \text{A} & \text{B} & \text{C} & \text{D} & \text{E} & \text{F} & \text{G}\\ 700 & \times & \times & 150 & 150 & \times & \times \end{array})$ if B died before C, which is different from $D_{\text{B}}\left(D_{\text{C}}P\right)=(\begin{array}{ccccccc} \text{A} & \text{B} & \text{C} & \text{D} & \text{E} & \text{F} & \text{G}\\ 500 & \times & \times & 250 & 250 & \times & \times \end{array})$, where B died after C. When we do not know the order of death for B and C, we cannot choose between $D_{\text{C}}\left(D_{\text{B}}P\right)$ and $D_{\text{B}}\left(D_{\text{C}}P\right)$. To solve this problem, we propose the commutativity criterion (C*):

(C*): The inheritance is commutative, meaning that if several persons died in the same event, the order of their deaths does not affect the result of inheritance. In other words, different operators $D_{\text{A}},D_{\text{B}}$ are interchangeable.

If this commutativity criterion (C*) is satisfied, then we do not need to care about the order of death problem. Nevertheless, in the next section, we shall prove that (C0), (R1)-(R3), and (C*) are incompatible. In the appendix, we present inheritance methods that satisfy (C0), (C*), two rules in (R1)-(R3), and three extra criteria (C1)-(C3). To satisfy (C*), we can even construct an inheritance method that breaks (C0): all the heritage goes to the oldest living person with the same family name as the deceased person. We do not aim at illustrating that (C0) and (R1)-(R3) are more desirable than (C*) with social scientific arguments. We just introduce inheritance methods resulting from breaking different requirements, and let social scientists and legislators determine which is better.

### 3.2 Assigning the order of death and the french approach

For an inheritance method that is not commutative, in some cases, when several persons died, the order of death affects the inheritance results. When the order of death is unknown, we need to make extra assumptions to compute the inheritance results. One choice is to artificially assign an order. However, assigning an order is not the only solution. At the death of a person, only living persons should be considered in inheritance. Therefore, the order of death for A and B helps to answer the following two questions: Can we confirm that A died after B, so that A should be considered in the inheritance of B? Can we confirm that B died after A, so that B should be considered in the inheritance of A? Notice that the negation of “we can confirm that A died after B” is “we cannot confirm that A died after B”, and in this case, A should be excluded from the inheritance of B. There are four possibilities regarding these two questions.

The first possibility is to assume that A might not die after B, and B died after A, so that A is excluded from the inheritance of B, and B is considered in the inheritance of A. This possibility is equivalent to assigning the order “A < B”.

Symmetrically, the second possibility is to assume that B might not die after A, and A died after B, so that B is excluded from the inheritance of A, and A is considered in the inheritance of B, which is equivalent to assigning the order “A > B”.

The third possibility is to assume that A died after B, and B died after A, so that A is considered in the inheritance of B, and B is considered in the inheritance of A. In this possibility, there is a direct contradiction, and we are trapped in an endless loop that A might inherit B, and B might inherit A. Therefore, the third possibility does not work.

The fourth possibility is to assume that A might not die after B, and B might not die after A, so that A is excluded from the inheritance of B, and B is excluded from the inheritance of A. This assignment has two interpretations. One interpretation is to regard this as assigning that A and B died simultaneously (denoted as A = B). The other interpretation is to regard the death time of A and the death time of B as incomparable (denoted as A ≈ B), but not assigning a generalized order. These two interpretations have a difference: if A = B and B = C, then we must have A = C; if A ≈ B and B ≈ C, then we do not need to have A ≈ C. The reason is that simultaneity is transitive, but incomparability is not.

Define $D_{B\backslash A}$ as the death operator of B, which processes the inheritance by assuming A is already dead (so that the status of A is not affected). In the above example, $D_{C\backslash B}P=(\begin{array}{ccccccc} \text{A} & \text{B} & \text{C} & \text{D} & \text{E} & \text{F} & \text{G}\\ 500 & 400 & \times & 50 & 50 & \times & \times \end{array})$. We can see that $D_{B}D_{A\backslash B}=D_{A}D_{B\backslash A}$. If we assign *A* < *B*, then the inheritance result is $D_{\text{B}}\left(D_{\text{A}}P\right)$; if we assign *A* > *B*, then the inheritance result is $D_{\text{A}}\left(D_{\text{B}}P\right)$; if we assign *A* = *B* or *A* ≈ *B*, then the inheritance result is $D_{B}\left(D_{A\backslash B}P\right)=D_{A}\left(D_{B\backslash A}P\right)$.

In sum, when the order of death for A and B is unknown, we can assign a **generalized inheritance order (GIO)** (which extends the concept of order of death) to determine the inheritance result. There are four choices for the GIO: A < B, A > B, A = B, A ≈ B. If we only assign A < B or A > B, then we obtain a strict total order for all dead persons. If we further allow assigning A = B, then we obtain a generalized total order for all dead persons. For example, according to the current Chinese Civil Code, if A and B died in the same event, and they both have living successors, then we assign A < B if A is B’s father, and we assign A = B if A is B’s brother. The French Approach always assigns A ≈ B. We can also combine assigning the order of death and the French Approach, so that all four GIOs are possible.

When the order of death for A and B is unknown, the assigned GIO for A and B should be deterministic, and only depend on the status of A and B (e.g., the relative relationship between A and B), not on other persons. An assignment that satisfies this condition is called **independent**.

Assume several persons died, and we only partially know the order of death. If we assign an order to each pair of persons with unknown order, there might be a contradiction with the known order. For example, assume A, B, C died, so that (1) the order of death for A and B is unknown; (2) the order of death for B and C is unknown; (3) A died after C (A > C). If the assigned order for A and B is A < B or A = B, and the assigned order for B and C is B < C or B = C, then we should have A < C or A = C, contradicting the reality that A > C.

The partially known order of death is not totally unrealistic. For example, assume A, B, C were involved in a disaster. A was found badly injured, and died at 9 AM; C was found badly injured, and died at 8 AM; B was found dead, and the death time was inferred by a forensic doctor to be between 7 AM and 10 AM. Then we do not know the order for A and B, and the order for B and C, but we know A > C.

One might propose that persons who died within the same short period of time should be regarded as dying simultaneously, so as to exclude such pathological examples. For example, in some states of the United States, by the Uniform Simultaneous Death Act, two persons died within 120 hours do not inherit each other (120 hour rule) [8]. However, this leads to the same problem: what if the deaths of two persons are separated by around 120 hours (but we do not know exactly whether it is longer or shorter than 120 hours)?

Fix an inheritance method. Assume several persons died, and we only partially know the order of death. There are multiple total orders of death that are compatible with the known order. For example, if we only know that A > C, then the following total orders are compatible with reality: C < B < A, C < A < B, B < C < A. Assume these orders do not produce the same inheritance result under the given inheritance method, so that assigning the GIO is necessary. If the assigned GIO and the known order do not have contradictions, then we call this assignment **consistent** with respect to the given inheritance method. If the assignment is not consistent (e.g., assigning A < B and B = C that contradicts the reality A > C), then the inheritance results cannot be determined.

We stipulate two requirements (S1) and (S2) for the assignment method for the GIO.

(S1) The assignment method for the GIO is independent.
(S2) The assignment method for the GIO is consistent with respect to the given inheritance method.

There are many assignment methods satisfying (S1), such as ordering by relative relationship or age. The French Approach that always assigns A ≈ B satisfies (S1) and (S2), since it does not introduce new orders that might lead to contradictions, and it does not consider any other persons. In the next section, we shall prove that purely assigning an order of death that satisfies (S1) would fail (S2). In fact, assigning A < B, A > B, or A = B in any case (even with a mixture of assigning A ≈ B in other cases) would lead to inconsistency or dependence.

We do not argue that (S1) must be followed. For example, in China, the assignment method considers whether the deceased person has other living successors, thus violating (S1), although (S2) also fails. If we break (S1), we can assign strict orders (“><”) that satisfy (S2). When the order of death is partially known, we have a partial order on the finite set of persons. By the order-extension principle, for a partially ordered set, such as A < C and B < D, we can extend the partial order to be a total order, such as A < C < B < D, so that any two elements are comparable [26]. For all possible order extensions, we can choose the one where the youngest person is as close to the rear as possible. If there are still multiple possible order extensions, we can consider the second youngest person, and so on. Since the assigned orders depend on all persons, (S1) does not hold. In general, if we assign an order of death that satisfies (S2), then (S1) is broken, and the assignment has to be much more complicated than the French Approach. We doubt whether such complicated assignment methods are suitable for being written into inheritance laws. We argue that the consistency requirement (S2) should be satisfied, since it guarantees the inheritance law to be complete.

When we process the inheritance for multiple persons, we should not process the inheritance of A before B, if A died after B and inherited B’s heritage. The order of death (whether from reality or from an assignment) determines a natural order of processing. We can process from the first deceased person to the last. If some persons are assigned to die simultaneously, since they do not inherit each other, we can choose any processing order. If we assign A ≈ B in some cases, then the dead persons are partially ordered. We can choose any compatible total order, since they produce the same inheritance result.

## 4 Impossibility results

With the preparations in Section 3, we shall prove that under some basic requirements, constructing a commutative inheritance method or assigning an order of death cannot solve the order of death problem. Under (C0) and (R1)-(R3), an inheritance method cannot satisfy the commutativity criterion (C*). With an inheritance method that satisfies (C0) and (R1)-(R3), the only assignment method for the GIO that satisfies (S1) and (S2) is the French Approach.

The results in this section shall be proved with the following example.

**Example 1**. *Consider a female A, A’s parents B and C, B’s parents D and E, C’s parents F and G. A, B, and C have no other sibling, descendant, or spouse. D, E, F, and G are alive. A, B, and C died in the same incident*.

### 4.1 Impossibility about commutative inheritance method

**Theorem 1**. Under (C0) and (R1)-(R3), any inheritance method fails the commutativity criterion (C*).

*Proof*. To prove this theorem, we just need to find two orders of death that produce different inheritance results. In fact, we shall prove a stronger statement: for all six possible strict total orders for A, B, C in Example 1, any three orders cannot produce the same inheritance result.

Based on different orders of death for A, B, and C, we check whether the following four statements about inheritance are true or not.

(I1) D and E can inherit all the property of B.
(I2) D and E can inherit some property of C.
(I3) F and G can inherit all the property of C.
(I4) F and G can inherit some property of B.

If the order of death is B < A < C, then at the death of B, due to (R1) and (R2), A can inherit some of B’s property. Due to (R1) and (R3), when A died, C can inherit some of A’s property. Due to (R1) and (R3), when C died, F, G can inherit all the property of C. Therefore, (I3) and (I4) are true, while (I1) and (I2) are false.

If the order of death is C < A < B, similarly, (I1) and (I2) are true, while (I3) and (I4) are false.

If the order of death is C < B < A or B < C < A, due to (R1) and (R2), A can inherit some of B’s property and C’s property. At the death of A, due to (C0) and (R1), all or none of D, E, F, G can inherit A. In either case, we can see that (I1) and (I3) are false.

If the order of death is A < B < C, then (I2) is false, and (I3) is true.

If the order of death is A < C < B, then (I4) is false, and (I1) is true.

When we compare the inheritance results for different orders of death, we can see that C < B < A and B < C < A are different from the other four possibilities. Besides, B < A < C is different from A < C < B, and A < B < C is different from C < A < B. Therefore, in these six orders of death, there are at least three different results, and any three orders cannot produce the same result. By (R1), if we replace A by a male, we still have the same results.

For an inheritance method that satisfies (C0) and (R1)-(R3), assume we only know the order of death for one pair of persons in A, B, C, and for the other two pairs, the order of death is unknown. (For example, we know A < B; we do not know the order for B and C; we do not know the order for A and C.) From the above proof, we can see that for the three possible total orders that are compatible with the reality (A < B < C, A < C < B, C < A < B are compatible with A < B), they cannot produce the same inheritance result. Therefore, we must assign a GIO for each unknown pair. If the assignment has a contradiction, then the consistency requirement (S2) fails.

### 4.2 Impossibility about assigning order of death

We introduce some notations to simplify the arguments. A(A=B) means that when the order of death for A and B is unknown, the assigned GIO is A = B. R(A<B) means that in reality, the order of death is A < B. (A∣B) means that the order of death for A and B is unknown. Thus a scenario [(A∣B),(B∣C),R(A<C)] means that the order of death for A and B is unknown; the order of death for B and C is unknown; the order of death for A and C is A < C.

About impossibility results in assigning GIO, we shall start with close relatives, then generalize to any relatives. Assigning A < B, A = B, A > B in any case would lead to contradictions. Thus we should always assign A ≈ B.

**Lemma 1**. *With any inheritance method that satisfies (C0) and (R1)-(R3), for A, B, C in Example 1, the only assignment for GIO that satisfies (S1) and (S2) is the French Approach*.

*Proof*. Choose any two persons in A, B, C, such as A and B. We will show that assigning A < B, A = B, or A > B would lead to contradictions in the order of death, and (S2) is violated. Thus A ≈ B is the only choice. With the same argument, we also have to assign A ≈ C and B ≈ C. The French Approach that always assigns A ≈ B introduces no new orders, thus no order contradictions. Therefore, it naturally satisfies (S2). Due to (S1), assigning the order of death for A and B is not related to C. Thus we can freely set when C died, such as “consider scenario [(A∣B),(B∣C),R(A<C)]” when A(A>B) and A(B>C).

**If** A(A>B) or A(A=B)

 **If** A(B>C) or A(B=C) or A(B≈C)

  Consider scenario [(A∣B),(B∣C),R(A<C)]

  R(A<C) and A(A>B) or A(A=B) lead to B < C, a contradiction

 **Else** A(B<C)

  **If** A(A>C) or A(A=C) or A(A≈C)

   Consider scenario [(A∣C),(B∣C),R(A<B)]

   R(A<B) and A(B<C) lead to A < C, a contradiction

  **Else** A(A<C)

   Consider scenario [(A∣B),(A∣C),R(B>C)]

   R(B>C) and A(A<C) lead to A < B, a contradiction

By switching “>” and “<” in the above proof, we can use the same argument to show that A(A<B) leads to a contradiction. Therefore, assigning A ≈ B is the only choice.

**Theorem 2**. *With any inheritance method that satisfies (C0) and (R1)-(R3), for any two persons D and E, the only assignment for GIO that satisfies (S1) and (S2) is the French Approach*.

*Proof*. Assume A(D>E) or A(D=E). If A(D<E), then we switch D and E. By Lemma 1, we can assume that D is not E’s parent, child, or spouse. Then we discuss depending on the relative relation between D and E. Similar to the proof of Lemma 1, by (S1), we can freely consider scenarios such as [(D∣E),(E∣G),R(D<G)], regardless of assigned orders.

(1) If D is not E’s parent-in-law, consider a family of E, E’s spouse F, E’s child G, E’s parents H and I, F’s parents J and K. Assume F is already dead, while H, I, J, K are alive. Since we exclude incest, D, E, F, G, H, I, J, K are different persons. Assume E, F, and G have no other living sibling, spouse, or descendant (possibly except D).

By Lemma 1, we have A(E≈G). Consider scenario [(D∣E),(E∣G),R(D<G)]. Then R(D<G) and A(D>E) or A(D=E) imply E < G, a contradiction.

Consider two orders of death, D < E < G and D < G < E, which are compatible with the reality D < G. In the proof of Theorem 1, we can see that the orders C < A < B and C < B < A produce different inheritance results. Thus if C is already dead, switching the death of A and B can affect the inheritance. At the death of D, we have reached the same situation. Thus D < E < G and D < G < E produce different inheritance results. Since different possible orders of death lead to different results, and the assigned GIOs have a contradiction, the consistency requirement (S2) is violated.

(2) If D is E’s parent-in-law, consider a family of E, E’s parents L and M, L’s parents N and O, M’s parents P and Q. Assume M is already dead, while N, O, P, Q are alive. Since we exclude incest, D, E, L, M, N, O, P, Q are different persons. Assume E, L, and M have no other living sibling, spouse, or descendant (possibly except D).

By Lemma 1, we have A(E≈L). Consider scenario [(D∣E),(E∣L),R(D<L)]. Then R(D<L) and A(D>E) or A(D=E) imply E < L, a contradiction.

Consider two orders of death, D < E < L and D < L < E, which are compatible with the reality D < L. With similar arguments, D < E < L and D < L < E produce different inheritance results. Since different possible orders of death lead to different results, and the assigned GIOs have a contradiction, the consistency requirement (S2) is violated.

Therefore, for any two persons D and E, assigning D ≈ E is the only choice. The French Approach that always assigns D ≈ E introduces no new orders, thus no order contradictions. Therefore, it naturally satisfies (S2).

To solve the order of death problem, there are three approaches: (1) construct a commutative inheritance method; (2) find a well-defined assignment for the order of death; (3) apply the French Approach that always assigns A ≈ B. Combining Theorem 1 and Theorem 2, we know that **under some basic requirements, the first and the second approaches (plus any mixture of the second and the third approaches) do not work. The French Approach is the only valid solution**.

**Remark 1**. *Certain inheritance laws avoid the order of death by executing the inheritance multiple times and taking average. For example, in the Minnesota State of the United States, when several persons died and the order of death is unknown, certain types of properties are inherited as follows: Find all possible orders of death. For each order of death, simulate the inheritance results. Finally, average over all these inheritance results to obtain the final inheritance results* [27].

*This method is not independent. Assume A died at 9 AM. The death time of B was inferred by a forensic doctor to be between 7 AM and 11 AM. Since A and B have equal probability to die before each other, it is fair to conduct inheritance for both A* > *B and B* > *A and take average. However, if C died at 10 AM, then there are three possible order of death: B* < *A* < *C, A* < *B* < *C, A* < *C* < *B. When we conduct inheritance for each of these orders and take average, we stipulate A* < *B in 2 of 3 cases, not one half. Thus C affects how A and B should inherit each other*.

*There is a variant of this idea that is independent: Assign a probability for each possible order of death. For example, in the above situation with A, B, C, we can assume the death time of B is uniformly distributed between 7 AM and 11 AM. Then with probability 1/2, B died before 9AM, and the order of death is B* < *A* < *C. With probability 1/4, B died between 9AM and 10AM, and the order of death is A* < *B* < *C. With probability 1/4, B died after 10AM, and the order of death is A* < *C* < *B. We conduct inheritance for each order of death and take weighted average for the inheritance results according to the probabilities. However, this method might be too complicated for an inheritance law, and it heavily depends on the forensic identification results, which can be disputable*.

**Remark 2**. *The current Chinese Civil Code stipulates an inheritance method that satisfies (C0) and (R1)-(R3). It also regulates how to assign a GIO when the order of death is unknown, although (S1) does not hold, since other relatives are considered. We shall show that the assignment also fails (S2)*.

*In Example 1, according to the current Chinese Civil Code, any two orders of death for A, B, C produce different inheritance results. Consider a scenario* [(A∣B),(B∣C),R(A<C)] . *Since* A(A>B), A(B=C), *we have A* > *C, a contradiction. Different orders of death that are compatible with the reality A* < *C produce different inheritance results. Thus the consistency requirement (S2) also fails. The current Chinese Civil Code does not regulate how to process such scenarios, although these scenarios are unlikely to happen in reality. In this sense, the current Chinese Civil Code is not well-defined*.

## 5 Conclusion and discussion

In many inheritance laws, when several persons died, and the order of death is unknown, the inheritance result cannot be determined. To solve this problem, there are two types of solutions (the second approach “assigning an order” and the third approach “the French Approach” are combined under the same framework).

(1) Construct an inheritance method that satisfies (C*). This means at least one of (C0) and (R1)-(R3) cannot hold. If (C0) fails, there are multiple inheritance methods that satisfy (C*). If we hold (C0) and (C*), there are four inheritance methods that satisfy two rules of (R1)-(R3) and three extra criteria (C1)-(C3). See the appendix for details.

(2) Apply an inheritance method that satisfies (C0) and (R1)-(R3), but not (C*). When the order of death is unknown, assign one GIO in “< = >≈”. If we require (S1) and (S2), then the only valid assignment method is always assigning “≈”, namely the French Approach. If we break (S1), there are multiple assignment methods, which are much more complicated than the French Approach. If (S2) does not hold, then the inheritance law is incomplete.

In sum, the only solution that satisfies (C0), (R1)-(R3), (S1), (S2) is the French Approach: whenever the order of death for two persons is unknown, stipulate that they do not inherit each other (each is assumed to die before the other) without assigning an order.

When we assign orders, (S2) fails only if the order of death for multiple persons is partially known, which is rare in reality. However, we should pursue well-defined laws. Besides, inheritance law is a highly theoretical and axiomatic subject, which should be rigorous. Therefore, we claim that (S2) must be followed, if the inheritance method does not satisfy (C*).

We do not argue that (C0) and (R1)-(R3) are more desirable than (C*), and we do not claim that (S1) must be satisfied when the inheritance method is not commutative. We just present the order of death problem and different solutions. This problem must be solved, and the inheritance law must be well-defined. We leave the work of arguing which solution is the most desirable to social scientists and legislators. Nevertheless, we personally appreciate the French Approach, since it is simple, understandable, and satisfies most requirements (except commutativity) in this paper.

This paper considers the intestate situation, where the deceased person left no will. Even if everyone has a will before death, there is still a possible problem about the order of death, since most wills are not commutative. Besides, in some countries, such as France and Germany, a portion of the heritage is forced to be inherited by law, not by will [25].

The area of inheritance law keeps evolving forever, and there should not exist an ultimate truth [28–31]. In the future, results in this paper might no longer be meaningful to the reality, although the mathematical proofs are still valid. In this era of new technologies, novel issues on inheritance law keep arising with developments in different fields, such as digital estate [32], artificial intelligence [33], and reproductive technology [34]. Specifically, big data bring in potential developments and challenges for inheritance law [35, 36]. For example, with empirical research from big data, a personalized default rule can diversify the inheritance results [37]. Besides, with the development of astronautics, when people can travel in the whole universe at nearly the speed of light, the special theory of relativity is in charge, and it is essentially difficult to determine the time order for two events (e.g., the death of two persons) happen in far away locations [38]. The failure to determine time-simultaneity might lead to a revolution of inheritance law.

## 6 Appendix: Commutative inheritance methods

We have shown that with (C0) and (R1)-(R3), an inheritance method cannot satisfy (C*). If we insist on (C*), can we have an inheritance method, at the cost of breaking some rules? The answer is yes. If we remove one rule from (R1)-(R3), the remaining criteria and rules are compatible. We will propose three more criteria (C1)-(C3) and show that there are finitely many inheritance methods that satisfy (C*), (C0)-(C3), and any two rules in (R1)-(R3).

We introduce some notions and then propose new criteria.

A “relative relation chain” between two relatives A and Z is a person sequence A, B, C,…, Y, Z, where neighboring persons have parent-child relation or marriage relation. For example, one relative relation chain between A’s child and A’s brother is: A’s child, A, A’s mother, A’s brother. There might be multiple relative relation chains between two relatives, such as full-blood siblings.

An “alive-dead configuration” describes the living/death status of each person.

“A can unconditionally inherit B” means that at B’s death, in any configuration, A can inherit B. For example, in general, children can unconditionally inherit parents, regardless of other relatives.

“A can conditionally inherit B” means that at B’s death, there exists a configuration, such that A can inherit B (meaning that some relatives are already dead or do not exist). Notice that “A can unconditionally inherit B” implies “A can conditionally inherit B”. For example, in general, grandparents can conditionally inherit grandchildren, if closer relatives are all dead.

Here are the three new criteria:

(C1) One person’s heritage should only be inherited by her/his spouse and blood relatives.
(C2) If every relative relation chain between A and B passes through another living person, then B cannot inherit A.
(C3) If a deceased person has living descendants that can conditionally inherit her/him, then her/his collateral relatives can not inherit.

Criterion (C2) is not always followed. In England and France, if the deceased person has no descendants, parents and siblings can both inherit [25], although siblings and the deceased person are blocked by parents.

We can show that there are four inheritance methods that satisfy (C*), (C0)-(C3), and two rules in (R1)-(R3).

**Proposition 1**. There is a unique inheritance method that satisfies (C*), (C0)-(C3), (R1), and (R2). This method is shown in Algorithm 1.
**Proposition 2**. There is a unique inheritance method that satisfies (C*), (C0)-(C3), (R1), and (R3). This method is shown in Algorithm 2.
**Proposition 3**. There are only two inheritance methods that satisfy (C*), (C0)-(C3), (R2), and (R3). These methods are shown in Algorithm 3.

The proofs of these propositions are at the end of this section.

There are two inheritance methods that satisfy (C*), (C0)-(C3), (R2), and (R3). In the first method, for a male, his inheritable descendants are son, son’s son, son’s son’s son, et al.; his inheritable ancestors are father, father’s father, father’s father’s father, et al; his inheritable collateral relatives are inheritable descendants of his inheritable ancestors; his wife is not inheritable. For a female, inheritable relatives are defined similarly by switching the gender, e.g., her daughter, mother, sister, et al. In the second method, for a male, his inheritable descendants are daughter, daughter’s son, daughter’s son’s daughter, et al.; his inheritable ancestors are mother, mother’s father, mother’s father’s mother, et al; his inheritable collateral relatives are inheritable descendants of his inheritable ancestors; his wife is not inheritable. For a female, inheritable relatives are defined similarly by switching the gender, e.g., her son, son’s daughter, father, father’s mother, sister, et al.

**Algorithm 1**: Detailed workflow of the inheritance method in Proposition 1.

1. **If** the deceased person has no living descendant

  **Inheritance**: heritage goes to the treasury

  **Terminate**

 **Else**

  **Inheritance**: her/his children (whether alive or dead) equally split all heritage

 **End** of if

2. **If** some children are already dead

  **Repeat** the inheritance procedure for each dead child

 **End** of if

**Algorithm 2**: Detailed workflow of the inheritance method in Proposition 2.

1. **If** the deceased person has no living ancestor

  **Inheritance**: heritage goes to the treasury

  **Terminate**

 **Else**

  **Inheritance**: her/his parents (whether alive or dead) equally split all heritage

 **End** of if

2. **If** some parents are already dead

  **Repeat** the inheritance procedure for each dead parent

 **End** of if

In all these four methods, the spouse cannot inherit. Besides, many other relatives are excluded from inheritance. The method in Proposition 1 excludes ancestors and collateral relatives; the method in Proposition 2 excludes descendants and collateral relatives; the two methods in Proposition 3 exclude half relatives depending on their genders. To incorporate criterion (C*), we have to limit the range of inheritability, so as to exclude various counterexamples. Here is the essence of the limitation: (C*) requires transitivity for inheritability: if A is an inheritable relative of B, and B is an inheritable relative of C, then A is an inheritable relative of C. Blood relative is not transitive: if A is a blood relative of B, and B is a blood relative of C, then A might not be a blood relative of C. These four methods that satisfy (C*) are transitive.

The method in Proposition 1 has a strange property: a child that died immediately after birth should also inherit, and her/his share would be transferred to the treasury. The method in Proposition 2 is generally inapplicable, since most people die after their ancestors, so that they do not have living inheritable relatives. The two methods in Proposition 3 are based on gender. To apply these two methods, one person must have a fixed gender, at most one mother and one father. Therefore, these two methods are ill-defined for LGBT+ groups, especially gay/lesbian couples with children.

### Preparations of proofs

Assume B died before A. If (C*) holds, we can assume that B was temporally resurrected right before the death of A. When the inheritance for A was finished, let B decease again and conducted the inheritance for B (the share inherited from A). Due to (C*), this “switching order of death” does not change the inheritance result. We shall apply this operation repeatedly.

**Algorithm 3**: Detailed workflow of the inheritance methods in Proposition 3.

1. **If** the deceased person has no living inheritable relative

  **Inheritance**: heritage goes to the treasury

  **Terminate**

 **Else**

  Eliminate all inheritable relatives who are already dead and have no living inheritable descendants

 **End** of if

2. **While** the deceased person has no living inheritable descendants

  **Inheritance**: her/his inheritable parent inherits all heritage

  **If** her/his inheritable parent is alive

   **Terminate**

  **Else**

   Eliminate this deceased person

   Regard her/his inheritable parent as the deceased person in this loop

  **End** of if

 **End** of while loop

 \\ After this loop, the considered deceased person has living inheritable descendants

3. **Inheritance**: all her/his inheritable children equally split all the heritage

 **If** an inheritable child is already dead

  **Repeat** this step for this inheritable child

 **End** of if

**Lemma 2**. Assume (C*) holds. Consider a configuration that A, B, C are alive. If B can inherit A in this configuration, then B can still inherit A if C is already dead.

*Proof*. In this configuration, assume A died before C. Then B can directly inherit A after A’s death. If A died after C, by (C*), we should still have the same result. Thus B can inherit A if C is already dead.

**Lemma 3**. Assume (C*) holds. Consider a configuration that A, B, C are alive. If B and C cannot inherit A in this configuration, then B still cannot inherit A if C is already dead.

*Proof*. In this configuration, assume A died before C. Then B cannot directly inherit A after A’s death. Since C cannot inherit A either, after C’s death, B cannot indirectly inherit A through C. If A died after C, by (C*), we should still have the same result. Thus B cannot inherit A if C is already dead.

Combining Lemma 2 and Lemma 3, we obtain the following corollary.

**Corollary 1**. Assume (C*) holds. Consider a configuration that A, B are alive. If we set C to be alive, and C cannot inherit A in this configuration, then we can freely set C to be alive or dead, without affecting whether B can inherit A.

**Lemma 4**. Assume (C*) holds. If A can conditionally inherit B, B can conditionally inherit C, then A can conditionally inherit C.

*Proof*. Assume A cannot conditionally inherit C. By Corollary 1, in a configuration P that B can directly inherit C, we can assume A is alive. Consider a configuration Q, where A can directly inherit B. Construct another configuration R, in which A, B, C are alive, and another person D is alive in R if and only if D is alive in both P and Q. Due to Lemma 2, in R, if C died before B, then B inherited C and A inherited B. If C died after B, by (C*), A should directly inherit C.

**Lemma 5**. Under (C*), (C0), and (C2), if in every relative relation chain between A and B, we can find a person C, such that C cannot conditionally inherit A, or B cannot conditionally inherit C, then B cannot conditionally inherit A.

*Proof*. Consider C_1_, …, C_*n*_ that C_*i*_ cannot conditionally inherit A, and consider ${\text{C}}_{1}^{\prime },\ldots ,{\text{C}}_{m}^{\prime }$ that B cannot conditionally inherit ${\text{C}}_{j}^{\prime }$. Here each C_*i*_ or ${\text{C}}_{j}^{\prime }$ is in a relative relation chain between A and B. If in a configuration, some C_*i*_ or ${\text{C}}_{j}^{\prime }$ are already dead, we can switch the order of death, so that A died first. At the death of A, due to (C2), B cannot inherit A, and C_1_, …, C_*n*_ cannot inherit A. Then at the death of ${\text{C}}_{j}^{\prime }$, B cannot inherit ${\text{C}}_{j}^{\prime }$, and by Lemma 4, C_1_, …, C_*n*_ cannot inherit ${\text{C}}_{j}^{\prime }$. At the death of C_*i*_, C_*i*_ cannot leave anything from A. Therefore, with the order that A died first, B cannot inherit A directly or indirectly. Even if some C_*i*_ or ${\text{C}}_{j}^{\prime }$ died before A, by (C*), B still cannot inherit A.

### Proof of Proposition 1

**Lemma 6**. Under (C*), (C0)-(C2), (R1), (R2), only descendants can conditionally inherit.

*Proof*. Consider Alice, her husband Bill, their daughter Clara, and Bill’s father David. By (R1) and (R2), Bill can unconditionally inherit David, and Clara can unconditionally inherit Bill. If parents can conditionally inherit children, then Alice can conditionally inherit Clara. By Lemma 4, Alice can conditionally inherit David, violating (C1). If spouses can conditionally inherit spouses, then Alice can conditionally inherit Bill. By Lemma 4, Alice can conditionally inherit David, violating (C1). By Lemma 5, collateral relatives and other ancestors, who are blocked by parents, cannot conditionally inherit. Since ancestors, collateral relatives, and spouses cannot conditionally inherit, by (C1), only descendants can inherit.

If the deceased person A has no living descendants, all heritage should go to the treasury, since there is no inheritable relative. If all children of the deceased person A are alive, then by (R1) and (C2), they equally split all the heritage. If a child B is already dead, we can switch the order of death, so that B inherited A, then died. Now we only need to repeat the inheritance for B. Notice that (C3) is not used in the proof. We can verify that this procedure, shown in Algorithm 1, satisfies (C*), (C0)-(C3), (R1), (R2). Besides, this procedure shall finish within finite steps.

### Proof of Proposition 2

**Lemma 7**. Under (C*), (C0)-(C2), (R1), (R3), only ancestors can conditionally inherit.

*Proof*. Consider Alice, her husband Bill, their daughter Clara, and Bill’s father David. Similar to the proof of Lemma 6, if children can conditionally inherit parents, or spouses can conditionally inherit spouses, then by (R1), (R3) and Lemma 4, David can conditionally inherit Alice, a contradiction. By Lemma 5, other descendants and collateral relatives cannot inherit. Since descendants, collateral relatives, and spouses cannot conditionally inherit, by (C1), only ancestors can inherit.

If the deceased person A has no living ancestors, since there is no inheritable relative, all heritage should go to the treasury. If both parents of the deceased person A are alive, then by (R1) and (C2), they equally split all the heritage. If a parent B is already dead, we can switch the order of death, so that B inherited A, then died. Now we only need to repeat the inheritance for B. Notice that (C3) is not used in the proof. We can verify that this procedure, shown in Algorithm 2, satisfies (C*), (C0)-(C3), (R1), (R3). Besides, this procedure shall finish within finite steps.

### Proof of Proposition 3

We start with many assumptions about who can inherit, and then check whether these assumptions are compatible with (C0)-(C3), (C*), (R2), and (R3).

(A1) Son can unconditionally inherit father.
(A1*) Son can conditionally inherit father.
(A2) Father can conditionally inherit son.
(A3) Daughter can unconditionally inherit mother.
(A3*) Daughter can conditionally inherit mother.
(A4) Mother can conditionally inherit daughter.
(A5) Son can unconditionally inherit mother.
(A5*) Son can conditionally inherit mother.
(A6) Mother can conditionally inherit son.
(A7) Daughter can unconditionally inherit father.
(A7*) Daughter can conditionally inherit father.
(A8) Father can conditionally inherit daughter.
(A9) Wife can conditionally inherit husband.
(A10) Husband can conditionally inherit wife.
(A11) Sister can conditionally inherit brother.
(A12) Brother can conditionally inherit sister.

**Lemma 8**. If (C0), (C1), (C*), (R2), and (R3) hold, then (A1)-(A4) are equivalent, and (A5)-(A8) are equivalent. If (A1)-(A4) hold, then (A1*) and (A3*) hold, (A5)-(A8), (A5*), and (A7*) fail. If (A1)-(A4) fail, then (A1*) and (A3*) fail, (A5)-(A8), (A5*) and (A7*) hold.

*Proof*. Consider a male Bill, his wife Alice, his son Charlie, his father David. If (A1) or (A1*) holds, and (A6) holds, then Bill can conditionally inherit David, Charlie can conditionally inherit Bill, Alice can conditionally inherit Charlie. By Lemma 4, Alice can conditionally inherit David, violating (C1). Thus (A1)/(A1*) and (A6) are contradicted. If (A5) or (A5*) holds, and (A2) holds, then Charlie can conditionally inherit Alice, Bill can conditionally inherit Charlie, David can conditionally inherit Bill. By Lemma 4, David can conditionally inherit Alice, violating (C1). Thus (A2) and (A5)/(A5*) are contradicted. If (A2) and (A10) both hold, then Bill can conditionally inherit Alice, David can conditionally inherit Bill. By Lemma 4, David can conditionally inherit Alice, violating (C1). Thus (A2) and (A10) are contradicted.

Consider a female Alice, her husband Bill, her daughter Clara, her mother Daisy. Using the same arguments (just switch the gender), we can show that (A3)/(A3*) and (A8) are contradicted, (A4) and (A7)/(A7*) are contradicted, (A4) and (A9) are contradicted.

Due to (R3), at least one of (A2) and (A6) holds; at least one of (A4) and (A8) holds.

Due to (R2), at least one of (A1) and (A7) holds; at least one of (A3) and (A5) holds.

Now we have two argument circles, where the notation ¬(A6) means (A6) does not hold:


$$\begin{array}{c} \text{(A1)}\Rightarrow \neg \text{(A6)}\Rightarrow \text{(A2)}\Rightarrow \neg \text{(A5)}\Rightarrow \text{(A3)}\Rightarrow \neg \text{(A8)}\Rightarrow \text{(A4)}\Rightarrow \neg \text{(A7)}\Rightarrow \text{(A1)};\\ \neg \text{(A1)}\Rightarrow \text{(A7)}\Rightarrow \neg \text{(A4)}\Rightarrow \text{(A8)}\Rightarrow \neg \text{(A3)}\Rightarrow \text{(A5)}\Rightarrow \neg \text{(A2)}\Rightarrow \text{(A6)}\Rightarrow \neg \text{(A1)}. \end{array}$$

We can see that (A1)-(A4) are equivalent, (A5)-(A8) are equivalent. If (A1)-(A4) hold, then (A1*) and (A3*) hold, (A5)-(A8), (A5*), and (A7*) fail. If (A1)-(A4) fail, since at least one of (A1) and (A7) holds, we have (A5)-(A8) hold. Then (A5*) and (A7*) hold, (A1*) and (A3*) fail.

**Lemma 9**. If (C0), (C1), (C*), (R2), and (R3) hold, then (A9) and (A10) fail.

*Proof*. Assume (C0), (C1), (C*), (R2), and (R3) hold. We have shown that (A2) and (A10) are contradicted, (A4) and (A9) are contradicted.

Consider a couple Alice and Bill, and Bill’s mother Daisy. If (A6) and (A10) both hold, then Bill can conditionally inherit Alice, Daisy can conditionally inherit Bill. By Lemma 4, Daisy can conditionally inherit Alice, violating (C1). Thus (A6) and (A10) are contradicted. Similarly, consider a couple Alice and Bill, and Alice’s father David, then we can show that (A8) and (A9) are contradicted.

Since (A2) and ¬(*A*6) are equivalent, (A10) is contradicted with both (A6) and ¬(*A*6). Thus (A10) can never hold. Similarly, (A9) can never hold.

**Lemma 10**. If (C0), (C1), (C*), (R2), and (R3) hold, then (A11) and (A12) fail.

*Proof*. Assume (C0), (C1), (C*), (R2), and (R3) hold. Consider a couple Alice and Bill, and their children Emily and Frank. If (A4) and (A11) both hold, then Emily can conditionally inherit Frank, Alice can conditionally inherit Emily. By Lemma 4, Alice can conditionally inherit Frank, satisfying (A6), which contradicts (A4). Thus (A4) and (A11) are contradicted. Similarly, If (A8) and (A11) both hold, then (A2) also holds, which contradicts (A8). Thus (A8) and (A11) are contradicted.

Since (A4) and ¬(*A*8) are equivalent, (A11) is contradicted with both (A8) and ¬(*A*8). Thus (A11) can never hold. Similarly, (A12) can never hold.

**Lemma 11**. Under (C0), (C3), (C*), and (R2), if a deceased person has living inheritable descendants, then her/his ancestors can not inherit.

*Proof*. Without loss of generality, assume the deceased person A is a male, and (A1) holds. Other scenarios can be treated symmetrically. Assume there exists a configuration, in which A has living inheritable descendants, and A’s father can inherit. By Corollary 1, we can assume A’s brother is alive. Then at the death of A’s father, by (R2), A’s brother can inherit A’s father. If we assume A’s father died before A, then by (C*), A’s brother can directly inherit A, violating (C3). Using the same argument, we can show that other ancestors (grandfather, great grandfather, et al.) cannot inherit.

From (C3) and Lemma 11, we can see that with living inheritable descendants, ancestors and collateral relatives cannot inherit. When the deceased person has no living inheritable descendants, and the inheritable parent (notice that only one parent can inherit) is alive, due to (C2) and Lemma 9, other relatives cannot inherit. Due to (R3) and Corollary 1, in this case, the inheritable parent must inherit.

Now we explicitly construct commutative inheritance methods. There are several different scenarios, and they could be mutually nested.

**Scenario (S0)**: The deceased person has no living inheritable relative.

If no one can inherit, the heritage goes to the treasury. Here the inheritance is finished.

**Scenario (S1)**: The deceased person A has living inheritable relatives, but does not have living inheritable descendants.

**Sub-scenario (S1.1)**: A’s inheritable parent B is alive.

In this case, the living inheritable parent B inherits all the heritage. Here the inheritance is finished.

**Sub-scenario (S1.2)**: A’s inheritable parent B is already dead.

We can apply (C*), and assume B died after A. Then B inherited A’s heritage, and we need to consider the inheritance for B. If B has living inheritable descendants, we go to scenario (S2) for B. If B has no living inheritable descendants, we go to scenario (S1) for B. If B’s inheritable parent C is also dead and has no living inheritable descendants, then we go to scenario (S1) for C. Since A has living inheritable relatives, this nesting of scenario (S1) shall stop within finite steps, and we either go to sub-scenario (S1.1) or go to scenario (S2).

**Scenario (S2)**: The deceased person A has living inheritable descendants.

**Sub-scenario (S2.1)**: All inheritable children of A are alive.

In this case, all inheritable children equally split all the heritage, since by (C2), their descendants cannot inherit.

**Sub-scenario (S2.2)**: Some inheritable children of A are already dead.

We can apply (C*), and assume A’s inheritable children died after A. Then these inheritable children equally split all the heritage, and we consider the inheritance problem for each child (if already dead). If the dead inheritable child B has living inheritable descendants, then we go to scenario (S2) for B. If the dead inheritable child C has no living inheritable descendants, then we go to scenario (S1.2) for C. By (C*), we assume that at C’s death, A was temporally resurrected and inherited C, and then deceased again. Then we go back to scenario (S2.2) for A. Here we seem to be trapped in an endless loop: A and C deceased iteratively, while C inheriting A, and A inheriting C. (A funny pun: in Chinese, “endless loop” literally means “dead loop”.) However, we have the following result, showing that the infinite iteration can be skipped by ignoring C. Thus the inheritance procedure can be finished within finite steps.

**Lemma 12**. Under (C0)-(C3), (C*), (R2), and (R3), if the deceased person A has a dead inheritable child C, who has no living inheritable descendants, then C and C’s descendants can be ignored in A’s inheritance.

*Proof*. If A has no living descendants, we go to scenario (S1), and C and C’s descendants are ignored. Otherwise, for simplicity, we assume that A only has three inheritable children, B_1_, B_2_ and C, where B_1_ and B_2_ have living inheritable descendants. At A’s death, B_1_, B_2_ and C each receives 1/3 of A’s heritage. Since C has no living inheritable descendants, C’s share (1/3) returns to A. By (C3) and Lemma 11, if B_1_ is dead, B_1_’s share will be distributed within B_1_’s inheritable descendants. The same applies for B_2_. Thus after the first iteration, A, B_1_ or B_1_’s descendants, and B_2_ or B_2_’s descendants each has 1/3 of A’s heritage. For the next iteration, the 1/3 portion by A is further split by B_1_, B_2_ and C. After the second iteration, B_1_ or B_1_’s descendants has 1/3 + 1/9 of A’s heritage, B_2_ or B_2_’s descendants also has 1/3 + 1/9, and A has 1/9. When the number of iteration goes to infinity, B_1_ or B_1_’s descendants has 1/3 + 1/9 + 1/27 + ⋯ = 1/2 of A’s heritage, and the same applies to B_2_ or B_2_’s descendants. This result is equivalent with the method that C and C’s descendants are ignored, and A’s heritage is directly split by B_1_ and B_2_.

The inheritance procedure is shown in Algorithm 3. It is complete: it considers all possibilities and finishes within finite steps. By Lemma 8, depending on whether (A1) holds, this procedure corresponds to two inheritance methods, differing by the definitions of inheritable relatives. If (A1) holds, a male’s inheritable relatives are those who share the same Y chromosome. A female’s inheritable relatives are those who share the same mitochondrial DNA. If (A1) does not hold, a male’s inheritable relatives are mother, mother’s father, brothers, daughters, daughter’s sons, et al. A female’s inheritable relatives are father, father’s mother, sisters, sons, son’s daughters, et al.

Our arguments show that there are no other methods that satisfy (C0)-(C3), (C*), (R2), and (R3). It is straightforward to verify that the two constructed methods satisfy (C0)-(C3), (R2), (R3), and Corollary 1. For the commutativity criterion (C*), consider a configuration that A and B are alive. If B cannot inherit A, then by Corollary 1, whether B died before or after A does not affect the inheritance of A’s property. If B can inherit A, from the construction of these methods, we can see that in a scenario that B died before A, these methods assume that B was temporally resurrected and inherited A, and then deceased again. Therefore, the case that B died before A is treated the same as the case that B died after A. Thus (C*) is also satisfied.

The above analysis depends on the assumption that one person has at most one father and one mother. Consider a lesbian couple with a son and a daughter. If (A3) and (A4) hold, then by (C*), the couple can inherit each other, contradicting Lemma 9; if (A3) and (A4) fail, meaning that (A5) and (A6) hold, then by (C*), the couple can inherit each other, contradicting Lemma 9. Therefore, an inheritance method that satisfies (C0), (C1), (C*), (R2), and (R3) cannot deal with gay/lesbian couples with children.
